# Melting Temperature Depression and Phase Transitions of Nitrate-Based Molten Salts in Nanoconfinement

**DOI:** 10.1021/acsomega.2c02536

**Published:** 2022-07-11

**Authors:** Mustafa Göktürk Yazlak, Qaiser Ali Khan, Martin Steinhart, Hatice Duran

**Affiliations:** † Department of Materials Science and Nanotechnology Engineering, TOBB University of Economics and Technology, Söğütözü Cad. 43, 06560 Ankara, Turkey; ‡ Institut für Chemie Neuer Materialien, Universitat Osnabrück, D-49069 Osnabrück, Germany; § UNAM Institute of Materials Science and Nanotechnology, Bilkent University, 06800 Ankara, Turkey

## Abstract

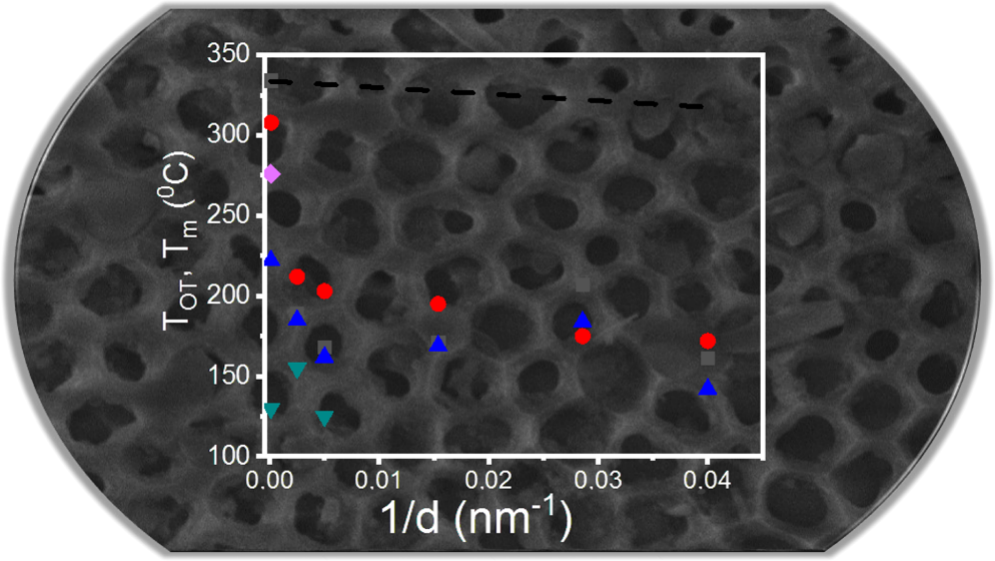

Hybrids of nitrate-based molten salts (KNO_3_, NaNO_3_, and Solar Salt) and anodic aluminum oxide (AAO) with various pore sizes (between 25 and 380 nm) were designed for concentrated solar power (CSP) plants to achieve low melting point (<200 °C) and high thermal conductivity (>1 W m^–1^ K^–1^). AAO pore surfaces were passivated with octadecyl phosphonic acid (ODPA), and the results were compared with as-anodized AAO. The change in phase transition temperatures and melting temperatures of salts was investigated as a function of pore diameter. Melting temperatures decreased for all salts inside AAO with different pore sizes while the highest melting temperature decrease (Δ*T* = 173 ± 2 °C) was observed for KNO_3_ filled in AAO with a pore diameter of 380 nm. Another nanoconfinement effect was observed in the crystal phases of the salts. The ferroelectric phase of KNO_3_ (γ-phase) formed at room temperature for KNO_3_/AAO hybrids with pore size larger than 35 nm. Thermal conductivity values of molten salt (MS)/AAO hybrids were obtained by thermal property analysis (TPS) at room temperature and above melting temperatures of the salts. The highest increase in thermal conductivity was observed as 73% for KNO_3_/AAO-35 nm. For NaNO_3_/AAO-380 nm hybrids, the thermal conductivity coefficient was 1.224 ± 0.019 at room temperature. To determine the capacity and efficiency of MS/AAO hybrids during the heat transfer process, the energy storage density per unit volume (J m^–3^) was calculated. The highest energy storage capacity was calculated as 2390 MJ m^–3^ for KNO_3_/AAO with a pore diameter of 400 nm. This value is approximately five times higher than that of bulk salt.

## 1 Introduction

There is an urgent need for the world to decarbonize energy supplies. Various options to do this include using wind power, classical photovoltaic-based solar power, and the emerging technology of concentrated solar power (CSP), whereby solar radiation is converted first to heat and then to work via a suitable thermodynamic cycle. They generate electrical power by concentrating the sun’s rays on the heat exchanger (receiver) mounted on the top of a tower. Thousands of sun-tracking mirrors, called heliostats, are used in these systems, which reflect the incoming sun rays. At around 290 °C in the solar power tower, a liquid heat transfer agent (HTA) is pumped toward the receiver in the cold storage tank, where the temperature is increased up to 560–570 °C and sent to the HTA hot storage tank.^1^ When power is drawn from the plant, the HTA is pumped into a steam generating system that produces superheated steam for a conventional turbine/generator system. The HTA in the steam generator returns to the cold tank, where it is stored and finally reheated in the receiver.

The thermal energy transferred from HTA is obtained by conventional means using Rankin cycles, Brayton cycles, or Stirling engines. Storage of thermal energy is as important as the efficient transport of heat energy in CSP facilities since heat storage systems allow for energy storage when electricity is not produced, thus providing electricity generation when there is insufficient sunlight. The general trend in the CSP plant design is that the fluid chosen as HTA is also suitable for use as a thermal energy storage (TES) material.

Molten salts and their mixtures have all of the desired properties for heat transfer agents (HTAs) and thermal energy storage (TES) fluids due to their low viscosity, low vapor pressure, low cost, high chemical stability, and environmental friendliness. Today, these melts are widely used as working fluids in nuclear power plants,^2^ the chemical industry,^3^ oil refineries,^4^ and concentrated solar power (CSP) plants.^5^ Choosing a suitable HTA and TES material for CSP is important to minimize the cost of solar receivers, thermal storage tanks, and heat exchangers and achieve high thermodynamic efficiency. Existing molten salt HTAs have high melting points (>200 °C) and begin to decompose above 600 °C. The main problem with this application is that salts can freeze easily in the evening or in winter, and in this case, it obstructs the working conditions by blocking the pipeline. Therefore, the development of inexpensive molten salt compositions with a lower melting point and higher thermal stability is important in improving both the thermodynamic efficiency of CSP plants and operating conditions.

Various materials such as water, thermal oil, and ionic liquids have been used as HTAs.^5^ Due to its low cost, high heat capacity, and high thermal conductivity, water is the first fluid that comes to mind as a HTA and TES fluid in many industrial applications. However, the very small temperature range in which the liquid state of water is preserved greatly restricts its use in CSP plants. Even in practical applications, the actual operating temperature is even lower than 100 °C, due to a large amount of material loss as it approaches the boiling temperature. While the usage of direct water vapor generally makes the system economical, the efficiency remains low as the operating temperature is restricted. The use of thermal oils as TES fluids in CSP plants is quite limited due to their disadvantages arising from some physicochemical properties. The upper temperature limit for these oils is about 300 °C, and above this temperature, the liquid state cannot be maintained. Moreover, the low thermal decomposition temperature, low density, and low heat capacity limit the potential of these oils in being used as HTAs. Although the melting temperatures are higher than that of water, the operating effective temperature range is still too narrow for practical applications. Ionic liquids are another type of fluid used as TES fluid. One of the main advantages compared to the other two liquids is that the liquid-state operating temperature range is much wider. In addition, their relatively higher chemical stability, low vapor pressures, high heat capacity, and low-density characteristics compared to water increase the thermal energy storage efficiency of ionic liquids. However, their ability to cause corrosion and high costs greatly limit the use of ionic liquids in energy transfer and storage.

Comparing the physicochemical properties and operating temperature ranges of all fluid classes listed above, molten salts were chosen as the working material in this study since they have optimum thermal conduction and thermal energy storage. The high heat capacity of these fluids, high thermal stability, and negligible vapor pressure increase their lifetime in CSP applications compared to other fluids. Generally, the liquid temperature range of pure molten salts is between 150 and 500 °C. This temperature range is also the operating temperature range of the heat storage device in which salt is used.

The method of energy storage using a low-melting-point salt solution is not only cost-effective but also environmentally friendly. De Luca et al.^6^ calculated and compared the power generation performance of a CSP plant and the cost of the system with and without salt solution TES. It was concluded that the annual electricity production was doubled when thermal storage was carried out using molten salts. Bellos et al.^7^ theoretically compared the thermal, hydraulic, and exergetic performances of thermal oil and salt solutions for CSP plants. It was calculated that molten salts showed higher exergetic performance compared to thermal oils. In the same study, the maximum exergetic efficiency for molten salt increased by up to about 38.4%. Furthermore, they reported the theoretical change in thermal and exergetic efficiency with the addition of CuO nanoparticles to thermal oil and salt solutions. The addition of CuO nanoparticles was calculated to increase the thermal and exergetic efficiency.

The most commonly used molten salts are potassium nitrate (KNO_3_) and sodium nitrate (NaNO_3_), with melting temperatures of 334 and 308 °C, respectively.^8,9^ However, despite all of their advantages, single-component nitrate salts do not meet the low melting temperature requirement. Therefore, multicomponent eutectic nitrate salt mixtures have been developed to meet the low melting temperature requirement. Eutectic mixtures provide stable and homogeneous thermophysical properties over the operating temperature range. The melting temperatures of multicomponent eutectic salt mixtures used in CSP plants are lower than 200 °C. Today, the most widely used multicomponent eutectic mixtures are solar salt (60 wt % NaNO_3_–40 wt % KNO_3_), Hitec (7 wt % NaNO_3_–53 wt % KNO_3_–40 wt % NaNO_2_), and Hitec XL (45 wt % KNO_3_–7 wt % NaNO_3_–48 wt % Ca(NO_3_)_2_). Among them, Solar Salt is the most commonly used salt mixture today. Although Solar Salt has the highest durability at high temperatures, it has the narrowest operating temperature range. The most important obstacle to increasing the working temperature of solar salt is the mass losses experienced at temperatures above 600–630 °C. Below these temperatures, the weight losses are relatively constant and can be successfully controlled, but above these temperatures, the mass losses begin to increase significantly. In contrast, multicomponent salts (ternary and quaternary mixtures) have lower melting temperatures.

To lower the melting point of conventional molten salts, Ren et al.^10^ developed a nitrate-based salt solution mixture (KNO_3_-NaNO_3_-LiNO_3_-Ca(NO_3_)_2_·H_2_O) (mass ratio, 6:1:2:2). The melting point of the mixture fell below 90 °C, and the reported average specific heat capacity was approximately 1.54 J g^–1^ K^–1^. With a similar approach, Zhang et al.^11^ prepared a salt mixture with LiNO_3_-NaNO_3_-KNO_3_-CsNO_3_. The melting temperature of this new mixture was reduced to 95 °C. In another study, by increasing the number of salt components (LiNO_3_-NaNO_3_-KNO_3_-KNO_3_-CsNO_3_-Ca(NO_3_)_2_) the melting temperature was reduced to 65 °C and the thermal decomposition temperature of the system was increased to over 500 °C.^12^ The addition of one or more LiNO_3_, CsNO_3_, and Ca(NO_3_)_2_ salts to its composition reduces the melting temperature of the resulting mixture, but LiNO_3_ and CsNO_3_ salts are expensive for large-scale usage.

Thermal conductivity is another critical heat transfer property for molten salts. Serrano-López et al.^3^ experimentally measured the heat capacity, thermal conductivity, and viscosity values of commercial molten salts. However, they reported that thermal conductivities were low in the operating temperature range (between 222 and 600 °C). Thermal conductivity of molten salts changes with temperature. However, some groups observed an increase in the thermal conductivity with temperature at high temperatures^13^ while some others reported that the thermal conductivity value of molten salts decreases with temperature at high temperatures.^14^ These contradictions arise mostly from (i) impurities and inhomogeneity, (ii) degradation of the salts at high temperatures, (iii) corrosion of the container material, (iv) the absence of a suitable temperature measurement device, and (iv) the role of heat transfer mechanisms such as convection and radiation at high temperatures.

Nanoparticles were extensively used to improve the thermal conductivities of the molten salts at both room temperature and high temperatures.^15−19^ SiO_2_, MgO, TiO_2_, and CuO nanoparticles were the most used oxides and SiC was the ceramic origin additive. Overall, the thermal conductivity of all salts increased by 5–20%. The source of enhancement was speculated as the addition of nanoparticles changes the structure of the salt melt and improves the heat transfer process in the mixture. The nanoparticles suspended inside the molten salts are under Brownian and similar forces, making irregular micromovements, which lead to microconventional movements and increase heat transmission. It is also stated that salt melt nanofluids have fractal-like flow structures so that the ionic components of salt melts cluster on nanoparticles, increasing thermal conductivity.^18^ On the other hand, the thermophysical properties of some samples worsened because of the heat treatments performed at high temperatures, thereby reducing thermal stability.^19^ The reduction of thermal stability seen in solar salt melt-based nanocomposites was related to the clustering of nanoparticles. Higher thermal stability was observed in samples with high–low temperature cycles compared to samples that remained consistently at high temperatures. However, in practical use, thermal cycling between low and high temperatures is inevitable and the segregation problem of nanoparticles should be solved in the long run.

Nanoporous anodic aluminum oxide (AAO) templates provide a two-dimensionally confined space in which self-organization processes such as crystallization can be fundamentally different from those obtained in thin films and in the bulk.^20,21^ A particular advantage of AAO templates is that they provide a range of parameter space (pore diameter, curvature, nature of pore walls) that can induce or manipulate self-assembly. Nowadays, a broad range of soft materials can be formed into nanorods by means of AAO templates containing arrays of self-ordered cylindrical nanopores.^22−25^ These templates are particularly suitable for the rational generation of nanofibers because equilibrium and nonequilibrium states and a range of unprecedented confinement-induced morphologies with new and exciting properties can be realized. Other important advantages of AAO templates are their homogeneous pore size distribution and good mechanical integrity. Abad et al.^26^ measured the thermal conductivity of AAO templates which varied between 1.03 and 1.32 W m^–1^ K^–1^ depending on the pore size. The same group filled the AAO pores with poly-(3-hexylthiophene) (P3HT) polymer^27^ and bismuth tellurium.^28^ They emphasized that the thermal conductivity of nanowires increases as the pore diameter increases, and it can be controlled by adjusting the orientation of the crystal inside the pores. The thermal conductivity of the bismuth tellurium/AAO composite was reported to be approximately 1.7 W m^–1^ K^–1^, a 31% increase. The advantages of AAO membranes are that (i) they do not allow for the segregation problem observed in nanoparticle salt mixtures and (ii) alumina minimizes corrosion frequently observed in metal parts in the high-temperature environments of molten salts.^29^ Although the thermal behavior of AAO and hybrid/composite structures of many organic and inorganic materials has been extensively studied in the literature, a limited number of studies have been reported for MS/AAO. Yadlovker and Berger^30,31^ carried out an XRD analysis of KNO_3_/AAO and observed that KNO_3_ crystals orient parallel to the longitudinal axis of the AAO pores. In another study,^32^ they studied potassium sodium tartrate salt grown in AAO pores (30 nm). They observed that the crystals have a monoclinic crystallographic structure and a smooth crystallographic orientation. The phase transitions of KNO_3_ in AAO are also investigated. An expansion was observed in the temperature range of the KNO_3_ ferroelectric phase for salts embedded in AAO pores with a pore diameter of 60 nm compared to the bulk salt.

Unfortunately, there is no study that systematically examines the effect of one-dimensional (1D) AAO nanostructure on the phase behavior, melting temperatures, thermal conductivity constants, and heat capacities of molten salts. In this study, we design a new nanostructured molten salt (MS) with a thermal conductivity constant >1 W m^–1^ K and melting point (<200 °C) to be used in CSP plants. Individual molten salts (NaNO_3_ or KNO_3_) and their binary mixtures (NaNO_3_/KNO_3_) were used as model salts.

## 2 Experimental Section

### 2.1 Materials

Potassium nitrate (≥99%), sodium nitrate (99%), octadecyl phosphonic acid (ODPA), *n*-heptane (99%), isopropyl alcohol (≥99.5%) copper(II) chloride dihydrate (CuCl_2_·2H_2_O) (≥99.0%), and hydrochloric acid (HCl) (≥37%) were purchased from Sigma-Aldrich.

### 2.2 Preparation of the Solar Salt (Eutectic) Mixture

It is very important to mix the salts homogeneously in the preparation of the eutectic mixture (solar salt, 60%NaNO_3_-40%KNO_3_). The following procedure was used to mix the salts homogeneously. NaNO_3_ and KNO_3_ were dried separately at 200 °C for 8 h. A salt mixture of 60 wt % NaNO_3_–40 wt % KNO_3_ by mass was prepared. Then, the mixtures were kept in molten form for 8 h at 400 °C. Afterward, these mixtures were left in the oven to cool to room temperature. The salt mixtures were then pounded with a mortar and pulverized. This process was repeated three times to ensure that the mixture was homogeneous.

### 2.3 Production of AAO Templates and Infiltration of Molten Salts into Nanopores

Self-ordered AAO templates (pore diameters of 25, 35, 65, 180, and 380 nm; pore depth of ∼100 μm) were fabricated by anodization of aluminum according to the procedure reported elsewhere.^32^ Membranes were kept in a vacuum oven (100 mbar) at 200 °C overnight. Before starting the infiltration process, the AAO templates were weighed five times on a precision balance (Mettler Toledo AX205 balance). Then, the nanopores were filled by applying the following procedure. A saturated aqueous salt solution was prepared at 70 °C, and then AAO templates were kept in an aqueous solution at 70 °C for 30 min. Afterward, MS/AAO was cooled from 70 to 25 °C at a rate of 0.25 °C min^–1^. Finally, salt-infiltrated AAO templates were removed from the solution at 25 °C and dried in a vacuum oven at room temperature overnight. The main purpose was to place the salt-water solution in the pores, start the cooling process, and then allow for salt crystals to grow in the pore. The salts remaining on top of AAO templates were removed with a soft emery cloth. This procedure was repeated until there were no salt residues left on the AAO surface in the scanning electron microscopy (SEM) images. To control the effective pore filling, AAO templates were reweighed after each infiltration process. When the weight remained constant, the procedure was terminated. The amount of salt in the AAO pores was determined from the weight difference between the filled and empty AAO membranes.

### 2.4 Material Characterization

#### 2.4.1 Differential Scanning Calorimetry (DSC) and Thermogravimetric Analysis (TGA)

Thermal analysis was carried out using a Mettler Toledo differential scanning calorimeter (DSC-822). Prior to DSC measurements, the Al substrates, to which the AAO membranes had been connected, were etched away by a solution of 100 mL of 37% HCl and 3.4 g of CuCl_2_·2H_2_O in 100 mL of deionized water at 0 °C and the samples were further milled to powder. Subsequently, 3–7 mg of sample material was sealed in aluminum pans (150 μL). DSC traces of water-infiltrated AAO were recorded using reference pans containing empty AAO pieces of the same pore diameter. All samples were first cooled at a rate of 10 °C min^–1^ from ambient temperature to −50 °C and then heated to 350 °C at the same rate under a N_2_ atmosphere.

Detection of the decomposition temperatures of bulk salts was carried out by thermogravimetric analysis (TGA) using a TGA Q500 device (TA Instruments) under a nitrogen flow (40 mL min^–1^) with a heating rate of 10 °C min^–1^ from room temperature to 900 °C. The temperature value at the point where the mass loss is 3% was considered as the decomposition temperature.

#### 2.4.2 Wide-Angle X-ray Scattering (WAXS)

The θ/2θ scans were taken with an XPERT-PRO (PANalytical, the Netherlands) diffractometer PW3050/60 with the sample stage MRD cradle configuration. The measurements were conducted with a step size of 0.02, and the total time for each measurement was about 14.5 h. The X-ray tube generator with a Cu anode operated at a voltage of 40 kV and a current of 40 mA. An aperture (divergence) slit of 0.25 mm was employed. A diffracted beam monochromator was inserted between the detector slit and the detector to suppress fluorescence radiation and the unwanted Kβ radiation. The Kα1 and Kα2 peaks could not be separated, and an average wavelength of 0.154251 nm was used. A PIXcel detector was used for all θ/2θ measurements. The AAO pore axes were oriented parallel and the AAO surface was oriented perpendicularly to the plane of the incident beam and the detector. In this geometry, only crystals that meet the Bragg condition and that have the corresponding set of lattice planes oriented parallel to the AAO surface can contribute to the scattered intensity.

#### 2.4.3 Scanning Electron Microscopy (SEM)

Scanning electron microscopy images of salt filling into AAO pores were obtained with a LEO Gemini 1530 device using 0.9–10 kV electron acceleration voltage.

#### 2.4.4 X-ray Photoelectron Spectroscopy (XPS)

To test the extent of surface functionalization of the inner surface of the AAO nanopores, surface scanning and high-resolution scanning of the appropriate elements were carried out. This analysis was performed by operating the Thermo Scientific K-Alpha X-ray photoelectron spectrometer sourced from Mg Kα (1253.6 eV) at 300 W and 117.40 eV transition energy. The spectra were recorded using a 60° take-off angle relative to the surface normal. The data analysis is conducted using Origin software. AAO surface composition data were calculated from full spectrum binding energy ranging from 0 to 1400 eV. High-resolution scans for C(1s), N(1s), and O(1s) were also performed for both raw and ODPA-coated membranes for comparison.

#### 2.4.5 Thermal Property Analysis (TPS)

The thermal conductivity values of the salt crystals confined to AAO pores were obtained by the hot disk transient plane welding (TPS) method at room temperature with the TPS 2500S hot disk thermal property analyzer. In the TPS method, the hot disc sensor consists of an electrically conductive pattern in the form of a double helix, etched with a thin metal (Nickel) foil. When performing a measurement, the hot disk sensor is placed between two pieces of the sample, each with the plane surface facing the sensor. The hot disk sensor is used both as a heat source and as a dynamic temperature sensor by applying an electrical current strong enough to increase the temperature of the sensor to a high temperature while simultaneously recording the increase in resistance (temperature) as a function of time. With this method, it is possible to obtain thermal conductivity values between 0.005 and 1800 W m^–1^ K^–1^ with an accuracy better than 3%.

## 3 Results and Discussion

**Figure 1:**
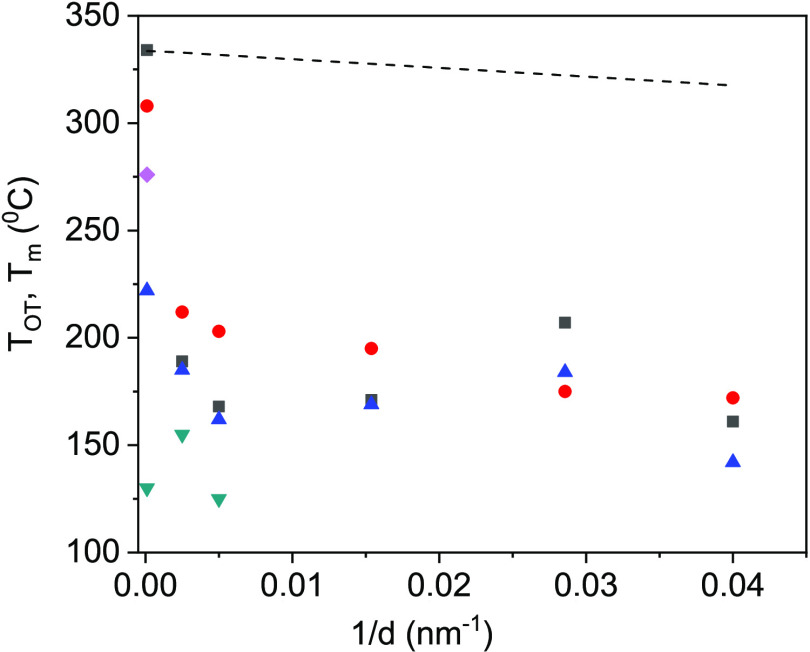
Phase diagrams of MS/AAO hybrids. (The dashed line is the Gibbs–Thomson relation fit to KNO_3_/AAO experimental data).

The AAO templates with small pores (25, 35, and 65 nm) were filled, whereas larger pores of AAO templates (180 and 380 nm) appeared to be partially filled. The melting temperature values of bulk salts (KNO_3_, NaNO_3_, and Solar Salt) and MS/AAO hybrids were measured by DSC (Figure S1). The melting temperatures of bulk salts and MS/AAO templates with different pore diameters are given in Table 1. Bulk KNO_3_ shows two peaks in DSC scans. The first endothermic peak maximum (130.4 ± 2.0 °C) corresponds to the crystallographic phase transition temperature (*T*_OT_) of KNO_3_, which is the transition from the orthorhombic (rhombic) crystal structure to the trigonal state.^33^ The second endothermic peak (peak temperature of 334.94 ± 3 °C) corresponds to the melting point (*T*_m_) of KNO_3_. Similarly, bulk NaNO_3_ had two endothermic peaks at 276 and 308 °C, which are the solid phase transition temperature (*T*_OT_) and solid melting (*T*_m_) temperatures, respectively.^34^ The melting temperature range of bulk solar salt is quite broad showing a peak maximum at 222 °C, which is very close to 220 °C reported in the literature.^35^ The melting temperatures of all MS/AAO composites decreased considerably compared to bulk salt. Although there is no systematic decrease in melting temperatures with decreasing pore diameter for all MS/AAO templates, a significant decrease is observed for all composite components when compared to the bulk (Figure 1). The highest decrease occurred for AAO with 380 nm pore sizes for all salts. It was also observed that the phase transition temperature (*T*_OT_) of KNO_3_ and NaNO_3_ disappeared when located in AAO templates. The melting temperature of KNO_3_/AAO composite salt decreases from the bulk value of 334–161 °C and that for the NaNO_3_/AAO composite, from 308 to 172 °C.

**Table 1:** Melting Temperatures of Bulk KNO_3_, NaNO_3_, and Solar Salt and MSs Filled in AAO Templates with Various Pore Diameters

	pore diameter [nm]	bulk	25	35	65	180	380
AAO parameters	interpore^a^ distance(nm)		71	96	117	350	595
	pore volume fraction^b^		0.057	0.111	0.141	0.145	0.580
melting temperature(°C)	KNO_3_/AAO	334	189	168	171	207	161
	NaNO_3_/AAO	308	212	203	195	175	172
	solar salt/AAO	222	185	162	169	184	142

a Interpore distance is the center-to-center distance between two pores.

b Pore volume fraction was calculated following Nielsch et al.^36^ from , where *R* is the pore diameter and *D*_int_ is the interpore distance.

In fact, the melting temperature drop inside the nanopores has been observed before for similar salts. Naberezhnov et al.^37,38^ obtained the maximum melting temperature drop of 40 °C for KNO3-filled nanoporous glass with a 7 nm pore size measured by dielectric spectroscopy. In another study,^39^ for porous glass with different pore diameters (320, 46, and 7 nm) filled with KNO_3_, it was observed that ferroelectric phase transition temperatures of the salt decreased with decreasing the glass pore diameters.

Confinement-induced melting temperature depression is a well-known phenomenon^40^ as it has been observed in other crystalline materials (polymers,^22,41,42^ liquid crystals,^43^ water,^44^ etc.). The magnitude of this decrease is less in semicrystal materials such as polymers (maximum Δ*T* ∼ 50 °C) because the ratios of the volume of the pore to the crystal nuclei are relatively closer to each other depending on the molecular weight of the polymers. However, the volume of salt crystals is much higher depending on supercooling.

The relationship between crystal size and melting temperature can be thermodynamically described by the Gibbs–Thomson relation having the form^41,45^
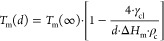
1where *T*_m_(∞) is the bulk melting temperature, *T*_m_(*d*) is the melting temperature of crystals within the pores with diameter *d*, γ_cl_ is the surface tension between crystal and liquid phases, Δ*H*_m_ is the bulk enthalpy of fusion, and ρ_c_ is the crystal density. From eq 1 using the values *T*_m_(∞) = 334 K, γ_KNO3_ = 0.110 N m^–1^,^46^ Δ*H*_m_ = 96.93 J g^–1^,^47^ and ρ_KNO3_^48^ = 2.11 g cm^–3^, the longitudinal dimension of the critical nucleus is calculated to be approximately 3 μm, which is consistent with the experimentally measured values.^46^ In fact, this size is much bigger than the diameter of the largest pores in this study. The volume of KNO_3_ bulk crystal^49^ is ∼10 × 10^–5^ mm^3^, whereas the volume of the 25 nm pore is ∼5 × 10^–11^ mm^3^ and the volume of the 380 nm pore is ∼1 × 10^–8^ mm^3^. There are 3–6 orders of magnitude difference between salt crystal and pore size. Since the probability of nucleation depends on the volume, and not on their surface area, salt crystals experience nanorestriction much more radically.

**Figure 2:**
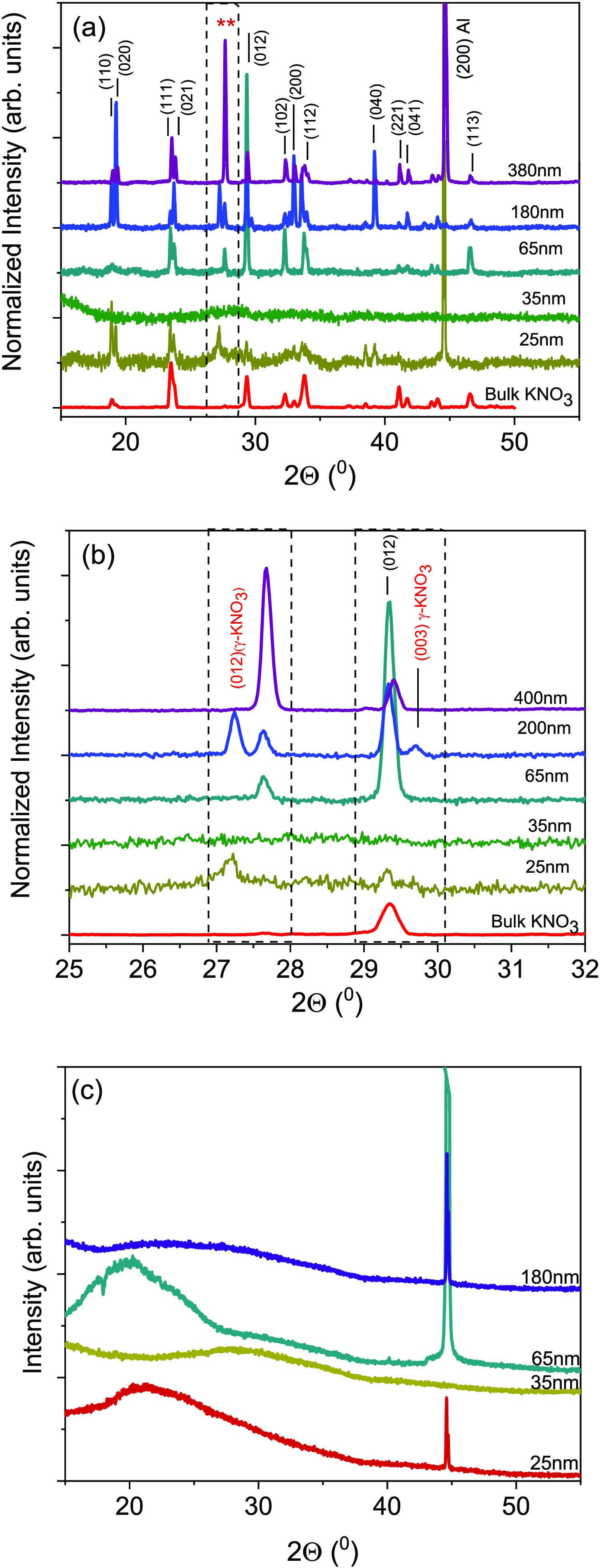
(a) Full-scale X-ray diffraction patterns of KNO_3_/AAO and (b) magnified (2θ between 25 and 32°) XRD patterns of KNO_3_/AAO and (c) ODPA-coated KNO_3_/AAO hybrids.

We also calculated melting temperature change as a function of pore diameter (dashed line, Figure 2). Eq 1 predicts a linear relationship between Δ*T*_m_ and 1/*d*. The theoretically calculated melting temperature does not fit the experimental measurements, although both show a decreasing trend. There may be several reasons for this difference between theory and experimental findings. Gibbs–Thomson equation would only be valid if the crystals have a smooth (i.e., nonfaceted) surface assuming an average solid–liquid interfacial energy. However, different crystal faces have different surface energies. As soon as particles are faceted, the effective surface energy depends on the relative weight of the different crystal faces forming a particle surface. Also, this relative weight may change during the melting process. In the case of cylindrical pores, strongly anisotropic crystal textures occur that may result in further departures from the Gibbs–Thomson model. Another reason might be the polymorphism of the crystals observed in the nanopores. More than one polymorph (α-phase, β-phase, and γ-phase of KNO_3_) is present in individual pores, and solid–solid transitions occur during heating. This might also result in the presence of small crystallites with dimensions smaller than the pore diameter so that the pore diameter may not be the geometrically determining confinement length. A similar deviation has also been reported in previous studies^41,50^

XRD analyses were performed at room temperature to evaluate the phases of the hybrids. For all data sets, the intensity was normalized to that of the (111) peak of α-phase since it is present in all samples. XRD revealed strong and sharp peaks at around 2θ = 44.5° arising from the aluminum substrate (Figure 2a). Bulk KNO_3_ has three phases at atmospheric pressure. The orthorhombic α-phase is stable at room temperature and transforms into the rhombohedral β-phase when heated above 130 °C.^51^ The X-ray patterns of bulk KNO_3_ indicated a diffraction peak of α-phase at 2θ = 23.48° and 23.76° of (111) and (021) reflections and 2θ = 29.34° and 33.70° of (012) and (112) reflections, respectively. When KNO_3_ is placed in the AAO pores, α-phase reflections were found for all pore diameters except for 35 nm. The (012) reflection of the ferroelectric γ-phase at 2θ = 27.3° and 27.67° was observed for pore sizes larger than 35 nm (Figure 2b). This form can exist only either at high pressures or on cooling from β-phase at 125 °C in the bulk and reverts to α-phase near 115 °C.^41^ Similarly, the formation of polymorphs, which are unstable under bulk conditions, in AAO has already been observed.^26,52−54^ Confinement effects, strain, deviation from stoichiometry, surface layer effects, and surface electric fields have been offered as mechanisms to stabilize thin-film ferroelectric crystal structures in thermodynamically unstable regions. Ferroelectric γ-phase of KNO_3_ was also observed in thin fused layers (75 μm) quenched to room temperature.^55^ Some theories predict that the stability of these phases is enhanced as the film thickness decreases, consistent with results on thin (1000 Å) vacuum-evaporated KNO_3_ films.^56^ It is reported that the ferroelectric γ-phase remains stable at temperatures below room temperature, but it is unclear whether this is a result of the size effect or the elastic stresses.^57^

Depending on the anodization conditions, the AAO pore walls may contain electrolyte anions, such as C_2_O_4_^2–^, PO_4_^3–^, and SO_4_^2–^ anions.^58,59^ These anions might interact electrostatically with salt solutions and affect the organization and orientation of salt crystals within the nanopore (interface effect). To distinguish this effect from the nanoconfinement effect, the pore surfaces were coated with ODPA to eliminate the ion effect arising from the production of AAO templates. In this way, we can investigate why molten salt infiltrated in 35 nm diameter AAO does not show a crystal structure, unlike the others. Surface modification of the AAO with a pore diameter of 35 nm was followed by XPS analysis (Figure S2). After the ODPA modification, the amount of carbon and phosphorus on the pore surfaces increased. For this sample, the occurrence of a phosphorus peak was considered qualitative proof of ODPA functionalization. It is observed that after pore passivation with ODPA, all crystal phase peaks disappeared regardless of pore dimensions (Figure 2c).

**Figure 3:**
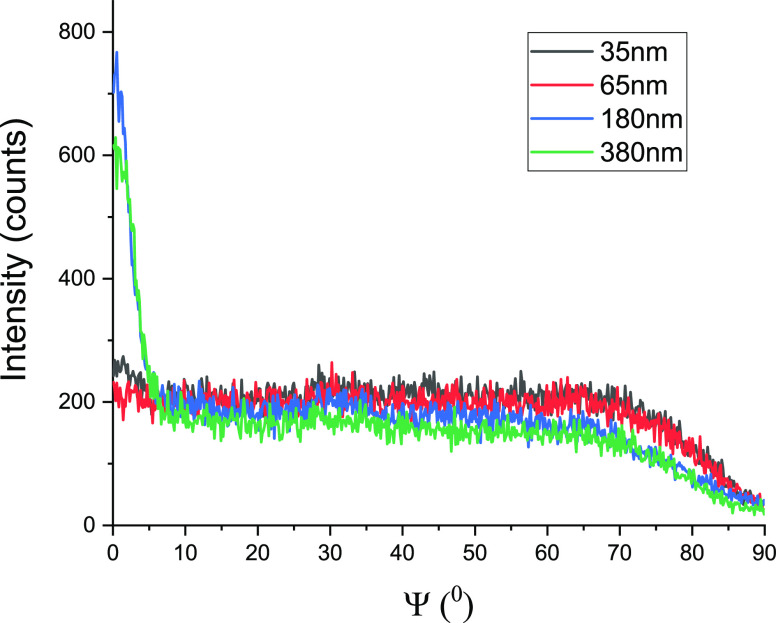
Azimuthal (ψ) scans of KNO_3_/AAO hybrids representing orientation distributions with respect to the AAO surface for the (012) lattice plane.

We measured azimuthal (ψ) scans for (012) Bragg reflection to analyze the distributions of the crystal orientations (Figure 3) in AAO templates. The orientation distributions of the (012) lattice planes show sharp maxima at ψ = 0° for AAO pore size larger than 35 nm. This indicates that there is a preferred orientation of the (012) lattice plane parallel to the AAO pore axes. Herman’s orientation parameter *S*, which quantifies the degree of orientation in anisotropic systems, is defined as follows
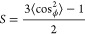
2where φ is the azimuthal angle and ⟨cos^2^ φ⟩ is the average calculated as
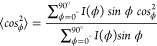
3where *I*(φ) is the intensity of the corresponding samples at a given azimuthal angle. For the molten salts infiltrated in pores with diameter smaller than 65 nm, there is no preferred alignment direction; therefore, *S* = 0, whereas the orientation factor *S* is around 0.34. When the pore size increases to 380 nm, the orientation factor *S* increases to 0.46.

The thermal conductivity measurements were taken at room temperature and above the melting temperature of each MS/AAO hybrid. Thermal conductivity values of MS/AAO obtained at room and high temperatures are given in Table 2. It is observed that the thermal conductivity values at high temperatures increase slightly compared to the measurements at room temperature (Table 2 and Figure S3).

**Table 2:** Thermal Conductivity Constants of Empty AAO and KNO_3_/AAO Composite Structures of Different Pore Diameters

pore diameter (nm)		25	35	65	180	380
thermal conductivity at 25 °C (W m^–1^ K^–1^)	empty AAO	0.485 ± 0.023	0.511 ± 0.019	0.498 ± 0.041	0.822 ± 0.027	0.967 ± 0.023
	KNO_3_/AAO	0.626 ± 0.027	0.822 ± 0.032	0.716 ± 0.063	0.957 ± 0.038	1.143 ± 0.027
	NaNO_3_/AAO	0.683 ± 0.019	0.834 ± 0.036	0.696 ± 0.057	1.153 ± 0.039	1.224 ± 0.019
	solar salt/AAO	0.543 ± 0.025	0.724 ± 0.033	0.603 ± 0.028	1.003 ± 0.051	1.107 ± 0.042
thermal conductivity at 300 °C (W m^–1^ K^–1^)	KNO_3_/AAO	0.696 ± 0.012	0.828 ± 0.019	0.733 ± 0.026	1.011 ± 0.017	1.213 ± 0.024
	NaNO_3_/AAO	0.740 ± 0.017	0.851 ± 0.028	0.710 ± 0.029	1.157 ± 0.021	1.265 ± 0.027
	solar salt/AAO	0.599 ± 0.017	0.752 ± 0.039	0.631 ± 0.037	1.067 ± 0.018	1.183 ± 0.034

The thermal conductivity values increase to a great extent by filling KNO_3_ into AAO templates with different pore diameters. The highest increase (∼73%) occurred for KNO_3_/AAO composite with a pore diameter of 35 nm. Similarly, the highest increase in thermal conductivity was observed in the NaNO_3_/AAO and Solar salt/AAO templates with a pore diameter of 35 nm. The prepared composite structures with pore sizes higher than 180 nm can be used as HTAs or TES materials in CSP plants since the thermal conductivity values increase greatly and a value above 1 W m^–1^ K^–1^ is obtained.

According to the thermal conductivity measurements taken at room temperature, the thermal conductivity values increased in all composite structures. According to DSC results, it was observed that the melting temperature decreased significantly for all hybrids. Therefore, the high-temperature thermal conductivity measurements conducted for the new melting temperatures were approximately 15 °C above the *T*_m_ value of each corresponding hybrid. The thermal conductivity value was the highest in the 25 nm KNO_3_/AAO composite with an increase of approximately 11% at 220 °C. Apart from this, the optimum Salt/AAO composite with a thermal conductivity coefficient *k* > 1 W m^–1^ K^–1^ is obtained for only 180 and 380 nm pore sizes.

Chliatzou et al.^13^ investigated the change of thermal conductivity constants of NaNO_3_ and KNO_3_ salts at high temperatures. They reported that the thermal conductivity value for both salts increased slightly at high temperatures compared to room temperature. On the other hand, Zhao et al.^29^ reported that the thermal conductivity value of eutectic salt at high temperatures decreased. In fact, when we look at the literature, it is seen that there are great inconsistencies between the data obtained from different researchers. One of the main reasons for these differences is the thermal conductivity measurement techniques used (transient hot-wire method, laser-flash method, etc.). When comparing the different methods available, the factors ignored in such measurements are (i) sample purity and homogeneity, (ii) thermal stability of salt, (iii) interaction between salt and the container material, (iv) the sensitivity of the measuring sensor, and (v) the presence of other simultaneous heat transfer mechanisms such as convection and radiation. Moreover, these two basic heat transfer mechanisms are neglected in theoretical studies to simplify the model. Another reason is that while one group of researchers performed their measurements under steady-state conditions,^60,61^ the other group performed them under unstable^62,63^ conditions. In fact, studies conducted with further thermal conductivity tests in recent years support our observation and show that the thermal conductivity constants of salt solutions do not change much with temperature.^3,5^

Energy storage density per unit volume (J m^–3^) is needed to determine the capacity and efficiency of MS/AAO composite structures in the heat transfer process. It was calculated according to the relation
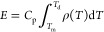
4

where *E* is the energy storage capacity, *C*_p_ is the specific heat capacity, *T*_m_ is the melting temperature, *T*_d_ is the decomposition temperature, and ρ is the density of MS/AAO calculated from eq 5. The AAO porosity values were obtained with the help of the ImageJ program using SEM images of empty AAO templates.

Effective medium theory (EMT) was used to calculate the effective density of empty and salt solution-filled AAO templates.
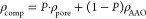
5

where ρ_pore_ denotes the density of the salts filled into the AAO pores, ρ_AAO_ denotes the skeleton AAO density, and *P* denotes the porosity ratio. *C*_p_ values of empty AAO, bulk KNO_3_, and KNO_3_/AAO hybrids were measured with DS, and the skeletal density of AAO membranes, and the densities of KNO_3_ salt in the pores, and the energy storage density calculated using eq 1 are given in Table 3. The energy storage density of bulk KNO_3_ is 480 MJ m^–3^.

**Table 3:** Specific Heat Capacity (*C*_p_), Effective Density (ρ), and Energy Storage Density (*E*) of KNO_3_/AAO Compositions

		*C*_p_ (J g^–1^K^–1^)		ρ (g cm^–3^)		
AAO (nm)	porosity(%)	empty AAO	KNO_3_	empty AAO	KNO_3_	*E*_KNO3_ (MJ m^–3^)
25	11	0.787	1.809	2.29	2.67	1879
35	12	0.857		1.98	2.59	1921
65	28	0.885	1.820	1.56	2.49	1844
180	29	0.878	2.026	1.03	2.36	1774
380	40	0.842	2.492	0.78	2.30	2390

An increasing trend in energy density with pore size (porosity) is observed. The highest energy storage capacity (∼2390 MJ m^–^^3^) was calculated for a pore diameter of 380 nm. This value is approximately five times higher than that of bulk salt. In general, it was observed that the energy storage density increased in composite structures compared to bulk salts. To be economically used as thermal energy storage materials, the energy capacity of these salts should be >756 MJ m^–3^.^5^ Clearly, KNO_3_/AAO hybrids have energy storage potential well above this limit value. Riazi et al.^64^ examined the technical and economic effects of improving the energy capacity of solar salt on CSP installations. Assuming the maximum potential specific heat increase of 200%, the volume of the heat storage medium decreased by 80%; the mass flow rate of the thermal fluid, the number of receiver tubes, and the pumping parasitic load decreased to 90%; and the net power output of the CSP tower increased to 3%. It is believed that a simple and inexpensive composite structure due to its advanced thermophysical properties will be a good alternative to expensive salt mixtures used in the energy industry.

## 4 Conclusions

Molten salts (KNO_3_, NaNO_3_) and eutectic mixtures of these salts were successfully infiltrated into the AAO pores. XRD analysis showed that the ferroelectric γ-phase of KNO_3_ forms a pore size larger than 35 nm. The ferroelectric salt crystals are oriented parallel to the AAO pore axes. Another significant observation was the decrease in the melting temperatures of all salts filled in AAO pores. The highest melting temperature decrease was observed in KNO_3_ salt filled in the 380 nm pore diameter. This drastic decrease in melting temperature has the potential to increase the operating efficiency of CSP plants. The melting temperature of the KNO_3_ salt decreased by a maximum of 173 °C. The highest thermal conductivity increase (∼73%) occurred for the 35 nm KNO_3_/AAO hybrid. These results clearly prove that the designed hybrid structures are ideal to be used as HTAs or TES materials in CSP plants. The prepared MS/AAO hybrid can be used not only in CSP plants but also in nuclear power plants due to the decrease in melting temperature, increase in thermal conductivity, and the strong mechanical integrity of AAO templates.

## Supporting Information Available

Melting temperature and thermal conductivity measurements of KNO_3_/AAO, NaNO_3_/AAO, and solar salts/AAO templates with various pore sizes and XPS spectra of AAO membranes modified with ODPA (PDF)
