# Supplementary material for: Melting Temperature Depression and Phase Transitions of Nitrate-Based Molten Salts in Nanoconfinement
Source: ACS Omega. 2022 Jul 11;7(28):24669–78. doi: 10.1021/acsomega.2c02536 (PMC9301948; doi:10.1021/acsomega.2c02536)
Supplement: Supplementary file 1 — ao2c02536_si_001.pdf [file ao2c02536_si_001.pdf]

Supporting Information for

Melting Temperature Depression and Phase Transitions of Nitrate-Based Molten Salts in Nanoconfinement

Mustafa Göktürk Yazlak[a], *Qaiser Ali Khan* [b], *Martin Steinhart* [b] *and Hatice Duran*[*,a,c]

[a] Department of Materials Science & Nanotechnology Engineering, TOBB University of Economics and Technology, Söğütözü Cad. 43, 06560 Ankara, Turkey

[b] Institut für Chemie Neuer Materialien, Universitat Osnabrück, D-49069 Osnabrück, Germany

[c] UNAM Institute of Materials Science and Nanotechnology, Bilkent University, Ankara 06800, Turkey.

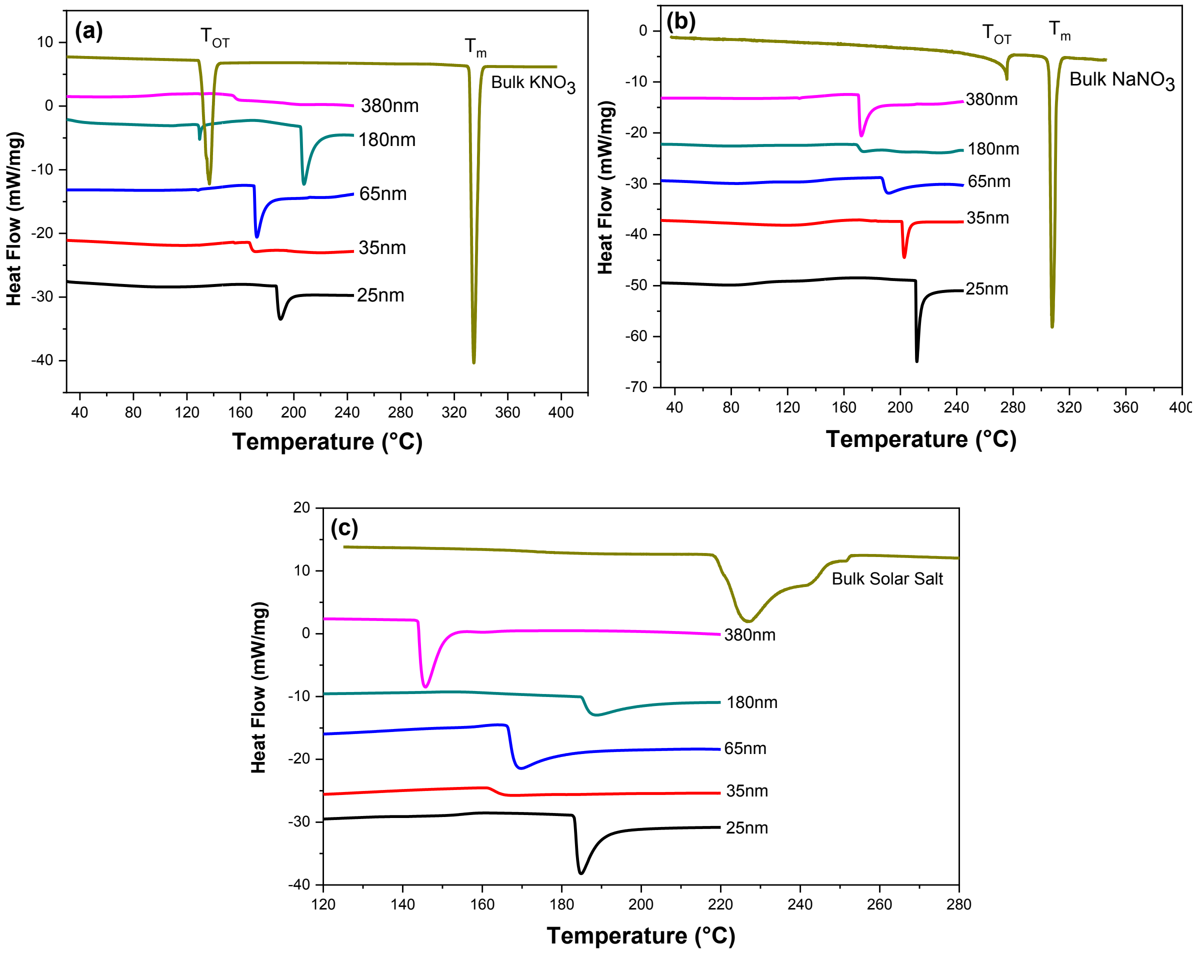

Figure S1: DSC measurements of (a) KNO_3, (b) $NaNO_3$, (c) Solar Salts infiltrated AAOs with 25, 35, 65, 180 and 380 nm pore diameters.

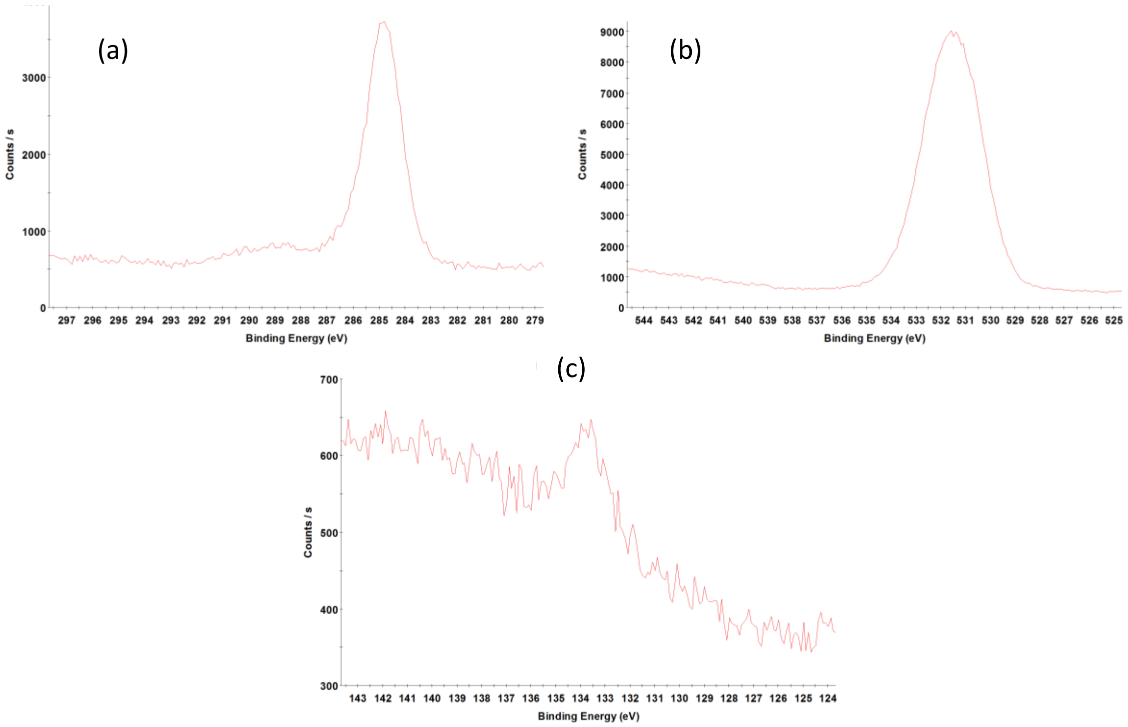

Figure S2: XPS high-resolution spectra for (a) C1s, (b) O1s and (c) P2p of AAO membranes with 35 nm diameter pores modified with ODPA

Table S1. Atomic percentages obtained from XPS on the surface of AAO membranes with 35 nm diameter pores.

	C 1s	O 1s	Al 2p	P 2p
**pristine AAO (35nm)**	10.94	53.09	35.96	0
**ODPA-AAO (35nm)**	29.56	43.67	24.77	1.27

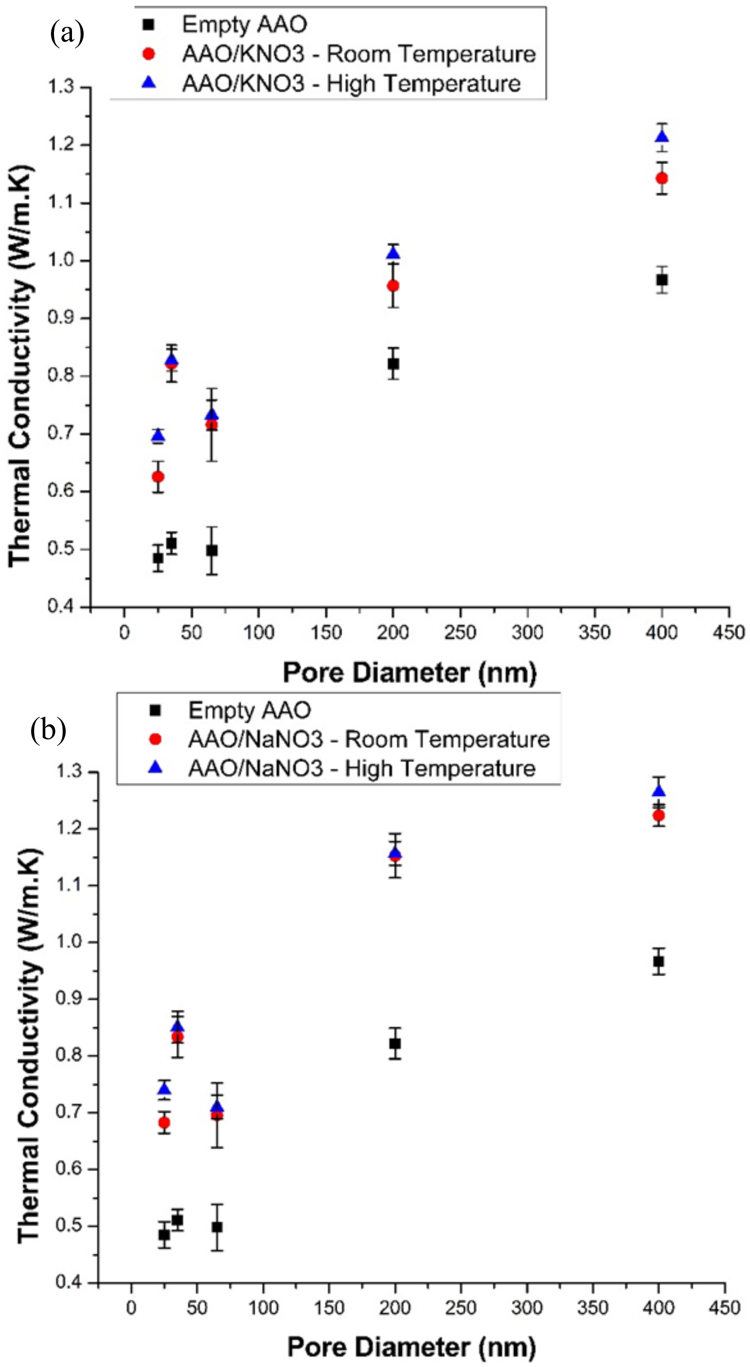

Figure S3: Thermal conductivity constants of (a) KNO_3/AAO and (b) $NaNO_3$/AAO with 25, 35, 65, 180 and 380 nm pore diameters at room (25 °C) and high temperature (300 °C).
